# Effectiveness of a brief psychotherapeutic intervention compared with treatment as usual for adolescent nonsuicidal self-injury: a single-centre, randomised controlled trial

**DOI:** 10.1007/s00787-019-01399-1

**Published:** 2019-09-11

**Authors:** Michael Kaess, Alexandra Edinger, Gloria Fischer-Waldschmidt, Peter Parzer, Romuald Brunner, Franz Resch

**Affiliations:** 1 University Hospital of Child and Adolescent Psychiatry and Psychotherapy, University of Bern, Stöckli, Bolligenstrasse 141c, 3000 Bern 60, Switzerland; 2 Section for Translational Psychobiology in Child and Adolescent Psychiatry, Clinic of Child and Adolescent Psychiatry, Centre for Psychosocial Medicine, University Hospital Heidelberg, Heidelberg, Germany; 3 Clinic of Child and Adolescent Psychiatry, Centre for Psychosocial Medicine, University Hospital Heidelberg, Heidelberg, Germany; 4 Clinic and Policlinic of Child and Adolescent Psychiatry, Psychosomatics and Psychotherapy, University of Regensburg, Regensburg District Hospital, Regensburg, Germany

**Keywords:** Nonsuicidal self-injury, Randomised controlled trial, Adolescents, Psychotherapy

## Abstract

Although nonsuicidal self-injury (NSSI) is a clinically significant behavior, evidence-based, specific, time-, and cost-effective treatment approaches are lacking. The aim of this study was to compare the efficacies of a brief cognitive-behavioral psychotherapy manual, the Cutting Down Programme (CDP), and treatment as usual (TAU) in the treatment of adolescent NSSI. We conducted a single-centre randomised controlled trial (RCT). Eligible participants were aged 12–17 years engaging in repetitive NSSI (at least 5 times within the past 6 months). We randomly allocated 74 participants to CDP (*n* = 37) or TAU (*n* = 37; in a 1:1 ratio). Outcome measures were administered before treatment (T0), directly after CDP or 4 months after baseline evaluation in the TAU group (T1), and another 6 months later (T2; primary endpoint). Primary outcome was a 50% reduction in NSSI frequency within the past 6 months at 10-month follow-up (T2). Regarding the primary outcome, there were no significant differences between the CDP (*n* = 26; 70.3%) and TAU group [*n* = 27; 73.0%; *χ*^2^(1) = 0.07; *p* = 0.797]; NSSI frequency within the past 6 months was significantly reduced at T2 [*χ*^2^(1) = 12.45; *p* < 0.001] with no between-group difference [*χ*^2^(1) = 0.14; *p* = 0.704]. However, we found a significant group x point of measurement interaction [*χ*^2^(2) = 7.78; *p* = 0.021] regarding NSSI within the last month indicating at T1. CDP was equally effective and achieved faster recovery compared to a significantly more intensive TAU in treating adolescent NSSI. The CDP could provide a brief and pragmatic first treatment within a stepped-care model for NSSI in routine clinical care.

*Clinical Trial Registration* The trial was prospectively registered in the German Registry of Clinical Trials (https://www.drks.de; DRKS00003605) and is now complete.

**Electronic supplementary material:**

The online version of this article (10.1007/s00787-019-01399-1) contains supplementary material, which is available to authorized users.

## Introduction

Nonsuicidal self-injury (NSSI) is defined “as the deliberate, self-inflicted damage of body tissue without suicidal intent and for purposes not socially or culturally sanctioned” (International Society for the Study of Self-Injury, ISSS). According to a large systematic review, approximately 17–18% of adolescents worldwide report at least a single episode of NSSI during lifetime [1]. NSSI is associated with a variety of psychiatric disorders and an elevated risk of suicidal behavior [2]

To date, there is no universally agreed best practice for the treatment of NSSI [3]. A recent systematic review revealed that there are effective treatments, which include or can be expanded to include the treatment of NSSI. However, these approaches were mostly neither developed for nor do they specifically focus on NSSI [4]. Particularly, in the context of (emerging) borderline personality disorder (BPD), NSSI is often treated with dialectical behavior therapy for adolescents (DBT-A), which was found to be effective within a randomised controlled trial (RCT) [5]. These treatment effects remained stable within an 1-year follow-up [6]. Other treatments that seem effective are mentalization-based treatment for adolescents (MBT-A) and cognitive-behavioral therapy (CBT) [3, 7].

The need for our trial is illustrated on the example of DBT-A: one of the first aims within the DBT-A target hierarchy is the elimination of severe NSSI. In further stages, however, DBT-A targets a broad spectrum of BPD symptoms beyond NSSI. Therefore, this approach is rather extensive. In addition, DBT-A requires intensive additional training for therapists. Hence, access to this treatment approach is restricted due to limited resources and a shortage of well-trained clinicians [8]. Considering the lack of such specialized treatments available as well as the transdiagnostic character of NSSI [9], less intensive and easy accessible treatments that address NSSI beyond BPD are essential. Such specific treatment approaches may improve the general standard of care, especially for adolescents who are often struggling to receive adequate professional help [10].

A randomised controlled pilot study investigated the effectiveness of a brief behavioral intervention for NSSI in young adults (Treatment for Self-injurious Behaviors, T-SIB) [11]. It was found that T-SIB was moderately effective for decreasing NSSI [11]. However, the study only enrolled a small number of participants, and did not focus on the critical period of adolescence [12].

In 1999, a cognitive-oriented and problem-focused short-term psychotherapy was developed for adults exhibiting nonsuicidal as well as suicidal self-injury [manual-assisted cognitive-behavior therapy (MACT)] [13]. Thus, it is important to note that this approach did not focus specifically on NSSI, such as T-SIB, but in addition on suicidal self-injury (SSI). Within a first RCT (*n* = 34), the subjects in the MACT group (*n* = 18) had a significantly greater reduction in the frequencies of self-harm incidents, suicide attempts and depressive symptoms compared to the treatment as usual (TAU) group (*n* = 16) [13]. However, within another large RCT (*n* = 480), there were no significant differences to a TAU, so that the use of the MACT was not supported in the routine treatment of patients [14].

In 2011, the MACT was adapted for adolescents in “The Cutting Down Programme” (CDP). A pilot study investigated 25 adolescents aged 12–18 years and provided preliminary evidence that the CDP may be efficacious in reducing NSSI, suicidality, comorbid depression, and trait anxiety [15]. Overall, 16 (64%) participants completed the intervention with an average number of only 8.5 sessions in this study. Outcomes were assessed at baseline, at the end of the treatment and at a follow-up after 3 months. Another important result was that the intervention was highly acceptable to patients and therapists. However, the sample size was rather small and the study did not employ a randomized controlled design [15].

In the present study, we tested the efficacy of the CDP compared to a more intensive TAU within a RCT. Our primary hypothesis was that significantly more adolescents in the CDP group would exhibit clinically significant reductions (at least 50%) in the frequency of NSSI within the last 6 months at follow-up (T2) than those in the TAU group. Primary outcome was a 50% reduction in the frequency of NSSI within the last 6 months at T2.

## Methods

### Study design

**Fig. 1:**
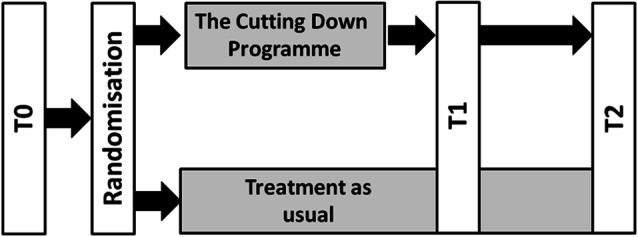
Study design

**Fig. 2:**
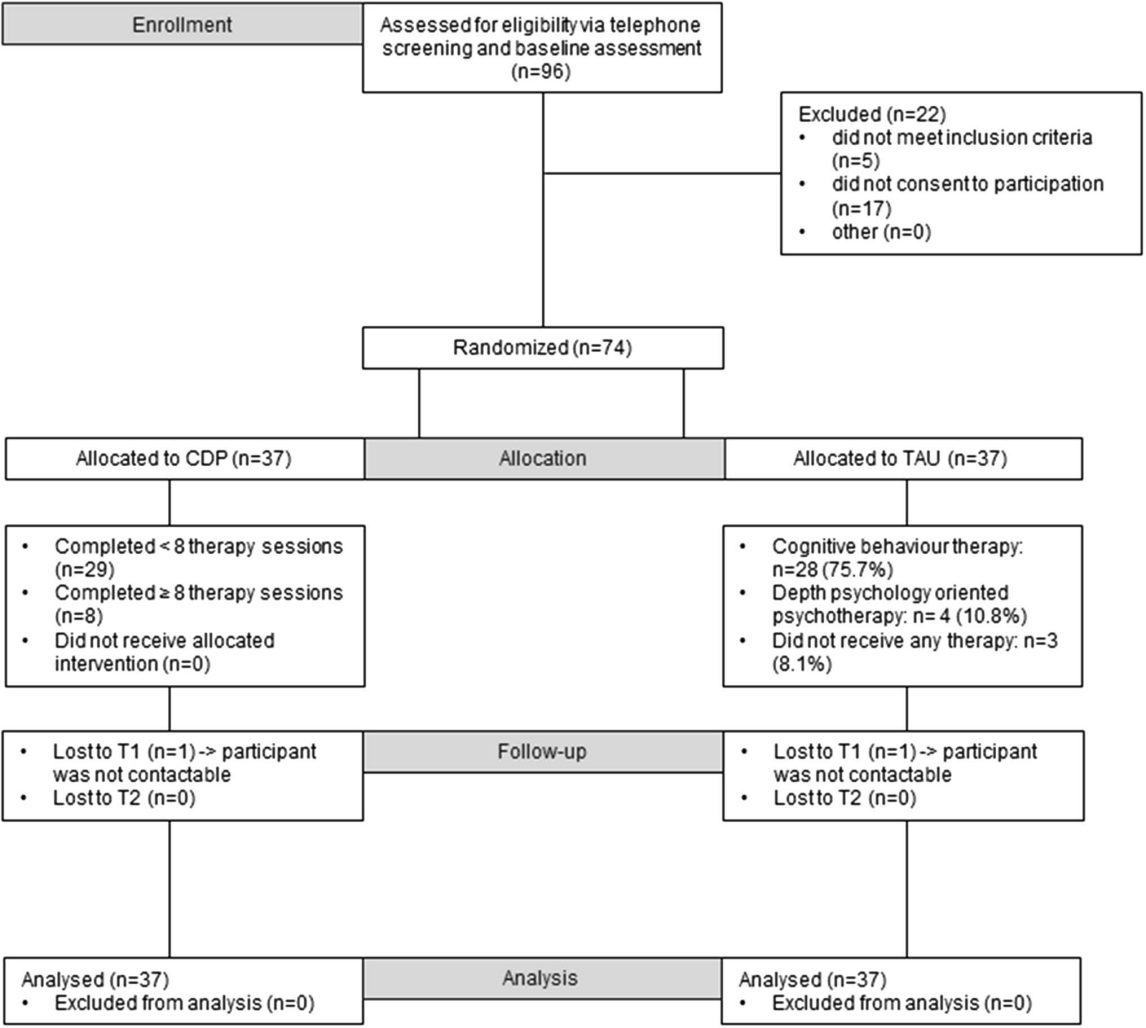
Trial profile

The present monocentric RCT aimed to compare CDP with TAU of higher treatment intensity. Figure 1 illustrates the study design, and Fig. 2 contains the trial profile. The RCT was conducted at the outpatient clinic for Adolescent Risk-taking and Self-harm behavior (AtR!Sk) at the Clinic of Child and Adolescent Psychiatry at the University Hospital Heidelberg, Germany. The authors assert that all procedures contributing to this work comply with the ethical standards of the relevant national and institutional committees on human experimentation and with the Declaration of Helsinki. All procedures involving human subjects/patients were approved by the institutional review board of the medical faculty at the University of Heidelberg (Ethics Committee No.: S-363/2011). The study design and procedures are presented in full in the published study protocol [16]. The trial was conducted and reported in accordance with CONSORT guidelines.

### Participant recruitment

Participants were recruited via our in- and outpatient units, or using official notices, newspaper articles, advertisements, and study flyers. The eligible participants were between 12 and 17 years, and they were required to have engaged in NSSI at least 5 days during the previous 6 months and at least once during the past month. Exclusion criteria were acute psychotic symptoms, acute intent to harm self or others that requires intensive psychiatric inpatient treatment, impaired intellectual functioning, and/or currently receiving psychotherapeutic treatment. Eligibility was established by a clinical psychologist during a telephone screening, and if adolescents were suitable, written informed consent was appropriately obtained from all participants and caregivers (if participants were below 16 years of age) either via mail or during an information appointment. Subsequently, participants were invited to attend a baseline assessment.

### Assessments

Participants were assessed at three timepoints: baseline (before randomisation, T0); after the completion of the CDP or 4 months after T0 within the TAU group to match the assessment points (T1); follow-up assessments (T2) 6 months after T1 (Fig. 2). To arrange for the T1 and T2 assessments, participants were contacted via phone. Participants received financial reimbursement following each assessment (T0–T2). All data were collected on paper forms and scanned for automatic data entry. Inconsistencies were resolved by checking the original paper material.

### Outcomes

The primary outcome was a 50% reduction of NSSI frequency within the past 6 months at T2, via the German version of the Self-Injurious Thoughts and Behaviors Interview (SITBI-G) [17, 18]. This interview has been validated in adolescents (12–19 years) [17] and is considered reliable (κs = 0.77–1.00) [18]. The choice to use a 50% reduction in NSSI as an indicator of efficacy was made, because it is a clinical relevant outcome. This procedure is derived from the approach of Jacobson and Truax [19] which relates to supplementary information, namely, clinically relevant information over and above the information available from test statistics [20].

The secondary outcomes were a 50% reduction of NSSI within the past month, changes in NSSI frequency over time (within the last month and the last 6 months), suicide attempts within the last 6 months, depressive symptoms, and quality of life. NSSI and suicide attempts were assessed at every timepoint (T0–T2) using the SITBI-G. The German version of the Beck-Depression-Inventory-II (BDI-II) [21] was used to assess depressive symptoms at T0, T1, and T2. The BDI-II is considered reliable (*r* = 0.93) [21], and adequate for the use in adolescent samples [22]. The KIDSCREEN-27 questionnaire for children and adolescents (8–18 years of age) was used to assess subjective health and well-being at T0, T1, and T2. This version shows good internal consistency (*α* = 0.70) [23].

Baseline assessment measures were comorbid mental disorders, which were assessed at T0 using the German version of the Mini-International Neuropsychiatric Interview for children and adolescents aged 6–17 years (M.I.N.I.-KID 6.0) [24, 25] and parts of the Structured Clinical Interview for DSM-IV-Axis II (SCID-II) [26]. In the present study, criteria for avoidant, dependent, borderline, and antisocial personality disorders were assessed. The M.I.N.I.-KID is a valid and reliable measure (κs = 0.64–1.00) [24]. Although the SCID-II has been validated in adults [26], it is suitable for the use in adolescents [27] and reliable (κs = 0.77–0.94) [28].

Within AtR!Sk, assessors underwent specific training and regular reliability checks. To check for inter-rater reliability, audiotaped interviews of each clinician, consisting of the M.I.N.I.-Kid [24], the SCID II (borderline, avoidant, dependent, and antisocial personality disorder) [26], as well as the SITBI-G [17] were recorded. Interviews were assessed by independent second raters blind for the first raters’ scores and diagnoses. Concerning the SITBI-G very good-to-perfect agreements were found within the inter-rater reliability checks (κs = 0.77–1.00) [18]. Regarding the SCID II, diagnostic agreement for full-threshold BPD was at 93.6% [29]. The clinician responsible for the interviews within the present study was involved in all inter-rater reliability checks mentioned in this section.

### Randomisation and masking

Participants were randomised after completing the baseline assessment (T0). They were randomly assigned (in a 1:1 ratio) to receive either CDP or TAU using baseline adaptive randomisation. The probability of assignment to the CDP group was set to the proportion of designated CDP participants in the not yet randomized participants. This prevents large differences in group size during the time of the study and reduces the influence of seasonal effects. The randomisation was conducted by a researcher who was not involved in the diagnostic or treatment procedures using pseudorandom numbers generated by Stata 12. The seed for the pseudorandom numbers was set to the time of day in seconds at the start of the randomisation. Allocation was undertaken using a password-protected website, independent of the trial. An independent staff member informed participants of the outcome of randomisation via telephone. All study assessments were performed by the same experienced and specially trained clinical psychologist at our clinic who was blinded to the participant’s group allocation for the duration of the follow-up period using a separate and encrypted patient list. When asked after completion of interviews which treatment the assessor thought each patient received initially, the assessor’s responses were correct for 49.1% of participants, indicating that masking was successful. Patients and clinicians were aware of treatment allocation.

### Treatment as usual

The participants in the TAU group were referred to local cooperating child and adolescent psychotherapists and psychiatrists either in private practice or in psychotherapeutic institutes. All participating therapists and institutes committed themselves to providing the first appointment and subsequent psychotherapeutic treatments within 2–4 weeks after baseline assessment. Psychotherapeutic approaches included either CBT or psychodynamic oriented psychotherapy. Most of the participants received CBT (*n* = 28, 75.7%; see Fig. 2). For detailed information on the TAU see Supplement 1.

### Cutting Down Programme

The manualized CDP that was applied in this study was based on elements of CBT and DBT and specifically tailored to the treatment of NSSI in adolescents. The treatment length was eight to 12 sessions. The manual organized the treatment into four modules that could be expanded with optional modules. Module 1 focused on promoting therapy motivation. The focus of module 2 was on identifying reasons for NSSI. In module 3, patients tested alternative behaviors to NSSI, and module 4 comprised stabilization of alternative behaviors [16]. The sessions were held once a week usually over a time of 2–4 months. Parental involvement was not specifically intended; however, appointments with parents were possible as needed. This decision was made by the therapist. Five therapists delivered 37 individual CDP therapies within our specialized outpatient clinic (AtR!Sk). For detailed information on the CDP see Supplement 2.

Study therapists were clinical psychologists (Master in clinical psychology) who were in training in CBT to become licensed psychotherapists. They already completed their intermediate exams after 1.5 years. In the context of the present study, they were trained in the CDP manual. Training consisted of two different aspects: in addition to the CDP treatment manual, there was a separate training manual to provide study therapists with detailed instructions and guidelines about the conduct of the treatment. Beyond that, an experienced clinical psychologist, who translated the manual, explicated the CDP training and treatment manual within a brief additional training session.

To measure adherence to the treatment manual, we used observational methods in terms of video recording and indirect measures. Video-based analyses of recorded therapy sessions were performed within supervision. An external supervisor, who was not involved in the study, checked the presented videos for adherence to the CDP manual. Types of rating were: occurrence of specific interventions (yes/no) and frequency counts (how often an intervention occurs, expressed numerically). This was done once in a month. In addition, indirect adherence measures were used. Thus, the study therapists documented the specific exercises of each module as well as the corresponding work sheets in standardized psychotherapy notes, which were checked once a week by an independent staff member.

### Statistical analysis

All analyses were intent-to-treat analyses (ITT). The study was powered to compare the effectiveness of the CDP and TAU in the treatment of NSSI. In secondary analyses, we used frequencies of NSSI in addition. We expected the CDP to result in greater reductions in NSSI incidents with a response rate difference of 35% (e.g. CDP: 60%; TAU: 25%). A response rate difference of 0.35 was considered to be clinically important. Similar rate differences were used within the power calculation of another RCT investigating CBT in patients with BPD [30]. To detect a rate difference of 0.35 with a power of 0.85 (*α* = 0.05), 35 participants were needed in each group.

Descriptive analyses were used to characterize the baseline study sample. Nominal data are presented as frequencies, while continuous data are presented as mean and standard deviation (SD) with confidence intervals. For variables with highly askew distribution, data are presented as medians and interquartile ranges. The influence of potential confounding variables was analyzed calculating regression models with and without covariates that were then compared using likelihood-ratio tests.

For our primary outcome, the TAU and CDP groups were compared with *χ*^2^ tests. Secondary outcomes were analyzed as follows. The changes in NSSI as well as in suicide attempts over time were analyzed with mixed-effects negative binomial regression because of the overdispersion of rates. In addition, to account for zero-inflation in suicide attempts, a zero-inflated negative binomial regression was calculated. To analyze a 50% reduction in NSSI within the last month, both groups were compared with *χ*^2^ tests. Changes in depressive symptoms and quality of life over time were analyzed with mixed-effects multi-level regression. All analyses were performed with Stata (version 14; Stata Corp LLC, College Station, TX, USA).

Data were monitored by an independent researcher who was not involved in the study procedures. The trial was prospectively registered in the German Registry of Clinical Trials (https://www.drks.de; DRKS00003605) and is now complete.

### Role of the funding source

The funder of the study had no role in study design, data collection, data analysis, data interpretation, or writing of the report. The corresponding author had full access to all the data in the study and had final responsibility for the decision to submit for publication.

## Results

Between July 1, 2012, and February 1, 2016, of 96 participants assessed for eligibility, we recruited 74 participants. Of these, 37 participants were allocated to receive CDP and 37 participants were allocated to receive TAU (Fig. 2). Primary and secondary outcome data were obtained for 37 (100%) participants in the CDP and 37 (100%) participants in the TAU group. We retained 74 (100%) of 74 participants over the 10-month follow-up period.

Baseline characteristics were balanced between the two groups (Table 1).

**Table 1:** Sociodemographic and clinical sample characteristics at T0

Sociodemographic variable/diagnostic category	TAU (*N* = 37)		CDP (*N* = 37)		Total (*N* = 74)	
Age	M	SD	M	SD	M	SD
	15.2	1.1	14.6	1.3	14.9	1.2
Sex	*N*	%	*N*	%	*N*	%
Female	34	91.9	37	100.0	71	96.0
Male	3	8.1	0	0.0	3	4.1
School type^a^	*N*	%	*N*	%	*N*	%
Hauptschule/foerderschule	3	8.1	8	21.6	11	14.9
Realschule	13	35.1	17	46.0	30	40.5
Gymnasium	21	56.8	12	32.4	33	44.6
Migration status	*N*	%	*N*	%	*N*	%
Kazakhstan	0	0.0	1	2.7	1	1.4
India	0	0.0	1	2.7	1	1.4
Russia	0	0.0	1	2.7	1	1.4
Spain	0	0.0	1	2.7	1	1·4
Portugal	1	2.7	0	0.0	1	1.4
Denmark	1	2.7	0	0.0	1	1.4
Germany	35	94.6	33	89.2	68	91.9
M.I.N.I.-Kid primary diagnoses^b^	*N*	%	*N*	%	*N*	%
No diagnosis	2	5.4	1	2.7	3	4.1
Current major depression	16	43.2	11	29.7	27	36.5
Past major depression	2	5.4	1	2.7	3	4.1
Recurrent depressive disorder	2	5.4	6	16.2	8	10.8
Dysthymia	9	24.3	7	18.9	16	21.6
Agoraphobia	1	2.7	0	0.0	1	1.4
Social phobias	1	2.7	1	2.7	2	2.7
Post-traumatic stress disorder	1	2.7	1	2.7	2	2.7
Drug/alcohol dependence	0 0.0		1 2.7		1 1.4	
ADHD	1	2.7	0	0.0	1	1.4
Oppositional defiant disorder	0	0.0	3	8.1	3	4.1
Affective disorders with psychotic features	0	0.0	1	2.7	1	1.4
Bulimia nervosa	0	0.0	1	2.7	1	1.4
Adjustment disorders	2	5.4	3	8.1	5	6.8
SCID-II						
Borderline personality disorder	8	21.6	15	40.5	23	31.1

*SD* standard deviation

^a^Foerderschule: school for students with special needs; Hauptschule: nine years of elementary school; Realschule: six years of school after four years of elementary school, terminating with a secondary school level-I certificate; Gymnasium: eight years of school after four years of elementary school, terminating with the general qualification for university entrance

^b^Multiple diagnoses per subject possible

Table 2 shows the number of completed therapy sessions as well as rates of supporting medication. The TAU group completed more therapeutic sessions on average than the CDP group did (*p* = 0.021), which indicates higher treatment intensity in the TAU group as expected. Concerning medication, there were no differences between the groups.

**Table 2:** Participation in intervention programs and effects on clinical outcomes

Intervention/clinical outcome	TAU		CDP		Group differences
Treatment adherence	M	SD	M	SD	*p *value^a^
Mean number of sessions attended to T1	5.0	4.3	9.6	2.7	< 0.001
Mean number of sessions attended to T2	14.3	13.0	3.3	6.5	< 0.001
Total mean number of sessions attended	19.3	14.0	12.9	7.9	0.021
Medication (subjects)	*N*		*N*		*P* value
T1					0.693
Antidepressants	2		1		
Neuroleptics	–		–		
Methylphenidate	–		1		
T2					0.258
Antidepressants	4		4		
Neuroleptics	–		1		
Methylphenidate	–		2		
NSSI in last 6 months	Median	IQR	Median	IQR	*p* value
					0.461
T0	60	30–95	50	25–90	
T2	8	1–50	10	2–40	
NSSI in last month	Median	IQR	Median	IQR	*p* value
				0.565	
T0	4	1–13	10	4–15	
T1	1	0–10	1	0–3	
T2	1	0–2	0	0–2	
Suicide attempts in last 6 months	M	SD	M	SD	*p* value
					0.353
T0	0.4	0.8	0.5	0.8	
T2	0.3	0.7	0.1	0.4	
BDI-II scores	M	SD	M	SD	*p* value
					0.980
T0	32.7	10.2	32.9	11.7	
T1	27.1	12.4	25.1	15.0	
T2	20.9	14.9	22.8	13.9	
KIDSCREEN-27	M	SD	M	SD	*p* value
					0.774
T0	38.1	5.4	39.0	7.0	
T1	41.0	6.2	42.6	7.5	
T2	44.7	8.4	43.7	8.9	

*IQR* interquartile ranges

^a^Group differences regarding primary and secondary outcome criteria

*Analysis of the primary outcome* showed that the majority of participants in both groups reached a 50% reduction in NSSI (TAU: *n* = 27; 73.0% vs. CDP: *n* = 26; 70.3%). Contrary to our hypothesis, there was no difference between the two groups [*χ*^2^(1) = 0.07; *p* = 0.797].

*50% NSSI reduction within the last month* Within the CDP group, significantly more participants (*n* = 28; 75.0%) exhibited reductions of at least 50% in the frequency of NSSI within the last month at T1 [*χ*^2^(1) = 4.25; *p* = 0.039] than the participants in the TAU group did (*n* = 19; 51.4%). At T2, the difference between the groups was no longer significant [*χ*^2^(1) = 2.09; *p* = 0.148].

**Fig. 3:**
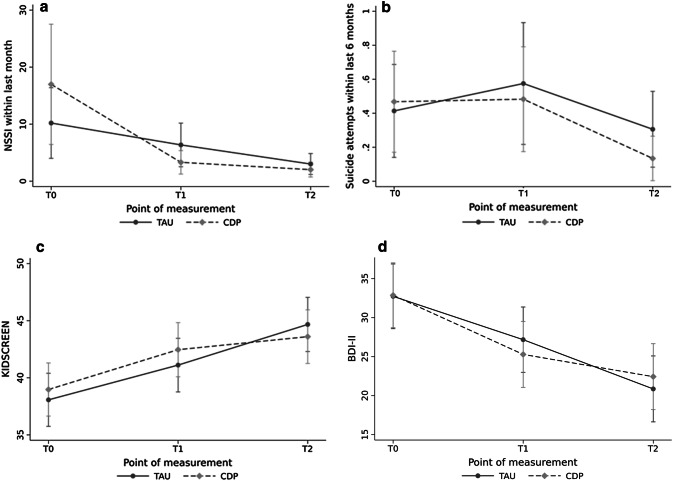
Secondary outcomes: change of NSSI frequencies within the last month, suicide attempts, quality of life, and depression

*NSSI frequencies within the last month and the last 6 months* Medians and interquartile ranges are shown in Table 2. Although both groups exhibited significant reductions in the frequency of NSSI incidents within the previous 6 months [*χ*^2^(1) = 12.45; *p* < 0.001], reductions did not differ significantly between the groups [*χ*^2^(1) = 0.14; *p* = 0.704]. Both groups also exhibited significant reductions in the NSSI frequencies within the last month [*χ*^2^(2) = 53.54; *p* < 0.001; see Fig. 3]. In addition, we found a significant group × point of measurement interaction [*χ*^2^(2) = 7.78; *p* = 0.021] regarding NSSI incidents within the last month. Thus, the CDP group exhibited a faster reduction in the frequency of NSSI incidents compared with the TAU group. This group × point of measurement interaction remained stable when controlling for the number of therapy sessions [*χ*^2^(2) = 8.77; *p* = 0.012].

Effect sizes for the reduction of NSSI frequency were large in both groups (TAU: Cohen’s *d* = 0.79; CDP: Cohen’s *d* = 0.99).

*Attempted suicides within the last 6 months* As shown in Table 2 and Fig. 3, both groups demonstrated decreased attempted suicides within the last 6 months over time [*χ*^2^(2) = 6.76; *p* = 0.034], with no significant difference between the treatment groups [*χ*^2^(1) = 0.86; *p* = 0.353]. This was also true when accounting for zero-inflation (Coef. − 0.57, 95% CI − 1.43 to 0.29, *p* = 0.195).

*Depressive symptoms* At T0, both groups were severely depressed (Table 2). As shown in Fig. 3, both the TAU and CDP groups reported significant reductions in depression over time [TAU: T0: 32.7; T1: 27.1; T2: 20.9; CDP: T0: 32.9; T1: 25.1; T2: 22.8; *χ*^2^(2) = 55.62; *p* < 0.001] with again no group difference [*χ*^2^(1) = 0.00; *p* = 0.980].

*Quality of life* The T0 assessment using the KIDSCREEN-27 showed that both groups had similar levels of well-being. As shown in Fig. 3 and Table 2, quality of life increased significantly for both groups over time [*χ*^2^(2) = 43.70;* p* < 0.001], without any group differences [*χ*^2^(1) = 0.08; *p* = 0.774].

There were no adverse events related to the interventions of the trial.

To control for potential confounding variables, we took treatment dose, BPD as well as depression as covariates within separate regression models into account, and compared them with the respective models without covariates. In general, models including covariates predicting the primary outcome did not differ from those without covariates (treatment dose: *p* = 0.851; BPD: *p* = 0.095; depression (BDI-II): *p* = 0.524). The same applied to the regression models that included the significant group × point of measurement interaction regarding NSSI incidents within the last month [treatment dose: *p* = 0.312; BPD: *p* = 0.407; depression (BDI-II): *p* = 0.142]. For further information on analyses of confounding variables see Supplement 3.

## Discussion

At T2, there was no evidence for the superiority of CDP compared with a significantly more intensive TAU for adolescents with repetitive NSSI. This was true for the primary outcome of a 50% reduction in NSSI frequency within the past 6 months as well as any of the secondary outcomes. Before this study, no study had compared the CDP with TAU.

Within a prior RCT with a 6-month follow-up, the adult version of the CDP, the MACT (*n* = 18), was compared to TAU (*n* = 16) and resulted in lower relapse rates regarding suicidal acts (56% for MACT vs. 71% for TAU) [13]. Beyond that, the CDP was investigated within a pilot study among 25 adolescents with repetitive NSSI. Results suggested preliminary evidence that the CDP may reduce NSSI, depression and trait anxiety [15].

The present findings showed that outpatient treatment with either CDP or TAU significantly and equally decreased NSSI, suicide attempts, and depression symptoms as well as significantly increased quality of life with large effect sizes at T2. These results are consistent with data showing that psychotherapeutic interventions are effective for treating NSSI [3]. T1 assessments, however, showed that the CDP group exhibited faster reductions in the frequency of NSSI within the last month. Results in the CDP group remained stable at T2 suggesting that the effects on NSSI endure even after termination of a brief intervention.

In our study, CDP patients received a significantly lower treatment dose (i.e., psychotherapy sessions) compared to the TAU patients. However, there was no effect of treatment dose on outcomes indicating that successful treatment of NSSI can be ensured using a brief psychotherapeutic intervention. Comparing our trial with previous studies, patients treated with MBT-A and DBT-A during previous RCTs had also completed significantly more therapeutic sessions compared to CDP patients who only completed a mean of 10 sessions [6, 7]. In addition, the TAU groups examined in other RCTs of NSSI provided a similar number of sessions compared to the respective index group [5, 7]. Our and other TAU groups were clinical samples with comparable group sizes [5, 7]. Regarding outcomes, however, the effect sizes for the reduction in the frequency of NSSI in previous TAU groups were rather small in the other RCTs (Cohen’s *d* between 0.23 and 0.40) [5, 7] compared to the large effect sizes found in the present study (Cohen’s *d* = 0.79). Finally, trials on both MBT-A and DBT-A revealed no superiority regarding celerity of NSSI reduction compared to the TAU groups [6, 7].

This RCT has high ecological validity concerning the majority of patients engaging in NSSI. Since NSSI can occur in the context of various disorders [12] and is commonly associated with suicidality [2], we did not exclude patients based on comorbid disorders or suicidality (except acute symptoms that prohibited outpatient treatment). Secondary analyses indicated that treatment effects were independent of major comorbid disorders [e.g., BPD or major depressive disorder (MDD)]. Thus, the CDP seems effective for treating NSSI in the context of a broad spectrum of comorbid diagnoses. Furthermore, our study focused on mid-adolescence, during which NSSI prevalence rates peak, while help-seeking is commonly low [10]. The internal validity of the trials was established through the fidelity of the CDP delivery, high rates of treatment adherence, perfect retention, and through masked outcome assessment. The external validity was maximized by eliminating waiting times within the TAU group and a 10-month follow-up.

This study had several limitations. First, because the sample predominantly consisted of female participants, conclusions cannot be made on possible gender differences. However, considering that the female gender has been identified as a risk factor for NSSI, the present sample depicted this finding [12]. Furthermore, we were not able to control for the naturalistic course of NSSI, because psychotherapeutic interventions are adequate and effective in treating NSSI [3]. Thus, it would not have been ethically defensible to allow participants to wait 10 months before receiving treatment. NSSI increases in early adolescence and decreases in late adolescence with a peak in mid-adolescence (15–16 years) [12]. Because our study sample had a mean age of 14.9 years (SD = 1.2) at T0, the frequency of NSSI might also have declined due to its natural course. In addition, the assumed rate difference between the CDP and TAU groups was rather optimistic. However, it should be noted that the group difference in response rate that we finally found in our trial was very small (2.7%); thus, a far larger sample would not have led to statistically differences in the main outcome.

In a rigorously conducted RCT, we have shown that CDP is not superior to TAU with higher treatment intensity over a 10-month follow-up for adolescent participants with repetitive NSSI but both treatments were associated with enduring positive outcomes in terms of a significant reduction in NSSI frequency, suicide attempts and depression as well as a significant improvement in quality of life. In addition, the CDP reached faster reductions in NSSI frequency compared to TAU. As a conclusion, the CDP showed similar treatment outcomes with a faster recovery while requiring less treatment sessions. This study provides important evidence that CDP might be an easily accessible, quickly available treatment for patients who would like an alternative to TAU. The brevity and potential cost-effectiveness of the CDP alongside its non-inferiority may nonetheless justify the implementation of such a brief psychotherapy manual in existing health care systems. As an example, the CDP could complement conventional interventions in terms of a stepped-care model to provide all concerned persons with easily accessible and quick professional help before performing more extensive treatments which can then be applied on those who really need them.

## Electronic supplementary material

Below is the link to the electronic supplementary material.

Supplementary file1 (PDF 265 kb)

Supplementary file2 (PDF 340 kb)

Supplementary file3 (PDF 294 kb)
