# Supplementary material for: Effectiveness of a brief psychotherapeutic intervention compared with treatment as usual for adolescent nonsuicidal self-injury: a single-centre, randomised controlled trial
Source: Eur Child Adolesc Psychiatry. 2019 Sep 11;29(6):881–91. doi: 10.1007/s00787-019-01399-1 (PMC7305262; doi:10.1007/s00787-019-01399-1)
Supplement: Supplementary file 1 — Supplementary file1 (PDF 265 kb) [file 787_2019_1399_MOESM1_ESM.pdf]

SUPPLEMENT 1

Title: Effectiveness of a brief psychotherapeutic intervention compared with treatment as usual for adolescent nonsuicidal self-injury – A single-centre, randomised controlled trial

Journal: European Child and Adolescent Psychiatry

Authors: Prof. Michael Kaess[1,2], M.Sc.-Psych. Alexandra Edinger[2], Dipl.-Psych. Gloria Fischer-Waldschmidt[3], Dipl.-Psych. Peter Parzer[3], Prof. Romuald Brunner[3,4], Prof. Franz Resch[3]

Corresponding author: Prof. Michael Kaess; University Hospital of Child and Adolescent Psychiatry and Psychotherapy, University of Bern, Stöckli, Bolligenstrasse 141c, 3000 Bern 60, Switzerland

Phone: +41 31 932 84 90; mailto: michael.kaess@upd.ch

Detailed information on the treatment as usual (TAU)

Organizational aspects

Participants within the TAU group received contact information of collaborating child and adolescent psychotherapists and psychiatrists in private practice or psychotherapy institutes. Our collaboration included two psychotherapy institutes located in Heidelberg, where psychotherapists were still in training but had already completed their intermediate exams. Here, psychotherapy was performed under continual internal and external supervision. Beyond that, we had collaboration with three private practices located in and around Heidelberg with already certified psychotherapists. In addition, collaboration included one registered specialist for child and adolescent psychiatry and psychotherapy.

There were three different therapy approaches within the TAU group: cognitive behavioral therapy, depth psychology oriented psychotherapy as well as pharmacotherapy. Most of the participants received cognitive behavioral therapy. All participating individuals and institutes committed themselves to providing the first appointment and subsequent psychotherapeutic treatments within two to four weeks after baseline. Usually, there are long waiting times (between three and 12 months) for child and adolescent outpatient treatment within the mental health care system. Participants usually received a regular appointment once a week.

Information on the education of psychotherapists in Germany

After five years of studies in psychology, students get a Master's Degree in (clinical) psychology after successfully completing their final examinations. Subsequently, they are allowed to start training in

psychotherapy which consists of initial 1.5 years of clinical work within a psychiatric clinic. Upon successful completion of a qualifying exam by the end of the first 1.5 years, the psychologists join the outpatient care where they have to complete 600 hours of closely supervised psychotherapeutic treatment. At the end of this time, they have to undergo state examinations to reach their “approbation”. Thus, psychotherapists in Germany undergo an extensive education.
