# Supplementary material for: Effectiveness of a brief psychotherapeutic intervention compared with treatment as usual for adolescent nonsuicidal self-injury: a single-centre, randomised controlled trial
Source: Eur Child Adolesc Psychiatry. 2019 Sep 11;29(6):881–91. doi: 10.1007/s00787-019-01399-1 (PMC7305262; doi:10.1007/s00787-019-01399-1)
Supplement: Supplementary file 2 — Supplementary file2 (PDF 340 kb) [file 787_2019_1399_MOESM2_ESM.pdf]

SUPPLEMENT 2

Title: **Effectiveness of a brief psychotherapeutic intervention compared with treatment as usual for adolescent nonsuicidal self-injury – A single-centre, randomised controlled trial**

Journal: **European Child and Adolescent Psychiatry**

Authors: **Prof. Michael Kaess[1,2], M.Sc.-Psych. Alexandra Edinger[2], Dipl.-Psych. Gloria Fischer-Waldschmidt[3], Dipl.-Psych. Peter Parzer[3], Prof. Romuald Brunner[3,4], Prof. Franz Resch[3]**

Corresponding author: Prof. Michael Kaess; University Hospital of Child and Adolescent Psychiatry and Psychotherapy, University of Bern, Stöckli, Bolligenstrasse 141c, 3000 Bern 60, Switzerland

Phone: +41 31 932 84 90; mailto: michael.kaess@upd.ch

Detailed information on the Cutting Down Programme (CDP)

Organizational aspects:

Participants received eight to 12 sessions of CDP. The sessions were held once a week usually over a time of two to four months. Parental involvement was not intended, however, appointments with parents were possible as needed. This decision was made by the therapist.

Content-related aspects:

The CDP is a cognitive behavioral therapy with components of DBT. The content is structured by a manual. Taylor et al. (2011) developed the manual by reviewing evidence base literature on the treatment of self-injurious behavior as well as maintenance factors and associated comorbidities. On that basis, adequate elements for the treatment modules of the CDP were identified. The final manual comprised of four modules:
(1) Getting Started; (2) Feelings, Thoughts and Behaviors; (3) Coping; and (4) On You Go

(1) Getting started

The first module is designed to take two to three sessions with an entirely optional session to promote motivation as needed. Aims of the module: a) to understand problems, strengths and goals of the patient; b) to give psychoeducative information on CBT and NSSI; c) to enable patients to identify and understand their feelings.

The first module comprises extensive psychoeducative information on NSSI including a cognitive behavioral model of NSSI. Afterwards, current problems and potential treatments goals are identified. In addition,

identification of already existing resources and interpersonal relationships is critical. An important part of the module is to introduce behavior analysis, which is first done with a fictitious patient and then transferred to the patient's own experiences. Finally, motivation and commitment for termination of NSSI is rated and, if needed, further promoted. As a last step, the module includes an introduction to the topic of "feelings", mainly containing psychoeducation.

(2) Feelings, Thoughts and Behaviors

This module is designed to take one to two sessions depending on how easily the patients understand the concepts. Aims of the module: a) to understand the links between thoughts, feelings and behaviors; b) to learn how to find a more helpful way of thinking.

The second module starts with the introduction of a mood, which then facilitates planning and implementation of particular helpful activities. In a next step, negative automatic thoughts as well as the help triangle (thoughts, feelings and behaviors in specific problematic situations) are introduced and adapted to the individual experiences. The next step includes psychoeducation on typical thinking errors (e.g. black and white thinking, overgeneralizing, etc.), which is then transferred to the patient's own problems and subsequently checked for reality. The final part of the module includes work on core beliefs and rules of living.

(3) Coping

The third module is designed to take three to four sessions. It includes strategies and skills that are based on the concepts of mindfulness and distress tolerance (adapted from DBT). Aim of the module: a) to enable the patient to explore a variety of coping strategies rather than NSSI.

In a first step, patients receive structured guidance on how to solve problems (identifying the problem, listing all possible solutions, discussing all solutions in terms of pros and contras, choosing the best solution, planning how to carry out the best solution and reviewing how to deal with obstacles). In a second step, patients learn more about assembling assertiveness, and are invited to practice given situations to become more assertive (e.g. disagree with someone's opinion). A further topic comprises "loving yourself", where self-critical thoughts are turned into helpful alternative thoughts. "Being mindful" is an introduction to mindfulness including the aim to balance "level-headed mind" and "feelings mind" to achieve a "sorted mind". Another important aspect is self-soothing when feeling distressed. Here, patients receive concrete stress tolerance skills to prevent NSSI.

(4) On you go

This module was designed to take one to two sessions. Aim of this module: a) to explore triggers for NSSI; b) to review coping strategies; c) to plan positive aspects of the immediate future.

The final module includes strategies to manage emotional crises and prevent a relapse. Patients anticipated possible events, feelings and thoughts that could push them to NSSI. Participants are encouraged to collect coping strategies for a crisis within a “first aid kit”. In another “tool box”, patients collect strategies to stay well and prevent a relapse. As a last exercise, participants identify positive things within the months to come.

Reference: Taylor LM, Oldershaw A, Richards C, et al (2011) Development and pilot evaluation of a manualized cognitive-behavioural treatment package for adolescent self-harm. Behav Cogn Psychother 39:619–625
