# Supplementary material for: Effectiveness of a brief psychotherapeutic intervention compared with treatment as usual for adolescent nonsuicidal self-injury: a single-centre, randomised controlled trial
Source: Eur Child Adolesc Psychiatry. 2019 Sep 11;29(6):881–91. doi: 10.1007/s00787-019-01399-1 (PMC7305262; doi:10.1007/s00787-019-01399-1)
Supplement: Supplementary file 3 — Supplementary file3 (PDF 294 kb) [file 787_2019_1399_MOESM3_ESM.pdf]

SUPPLEMENT 3

Title: **Effectiveness of a brief psychotherapeutic intervention compared with treatment as usual for adolescent nonsuicidal self-injury – A single-centre, randomised controlled trial**

Journal: **European Child and Adolescent Psychiatry**

Authors: **Prof. Michael Kaess[1,2], M.Sc.-Psych. Alexandra Edinger[2], Dipl.-Psych. Gloria Fischer-Waldschmidt[3], Dipl.-Psych. Peter Parzer[3], Prof. Romuald Brunner[3,4], Prof. Franz Resch[3]**

Corresponding author: Prof. Michael Kaess; University Hospital of Child and Adolescent Psychiatry and Psychotherapy, University of Bern, Stöckli, Bolligenstrasse 141c, 3000 Bern 60, Switzerland

Phone: +41 31 932 84 90; mailto: michael.kaess@upd.ch

Detailed information on further analyses

Control for confounding variables concerning primary outcome:

To control for potential confounding variables on the primary outcome, likelihood-ratio tests were performed. We considered treatment dose, BPD as well as depression as covariates within separate regression models.

In a first basic model, we considered the primary outcome criterion (a reduction of at least 50% in the frequency of NSSI within the previous six months at the T2 assessment; Table 1).

Table 1 Basic model

Treatment response	OR[a]	95% CI[b]	p-value
CDP	0.88	0.32-2.40	0.797

a OR=Odds Ratio
b CI=confidence interval

Examining treatment dose as a covariate, likelihood-ratio tests revealed that the model including treatment dose as a covariate and the model without treatment dose as a covariate did not differ: $\chi^2(2) = .32$; $p = .851$ (Table 2).

Table 2 Regression model with treatment dose as covariate

Treatment response	OR	95% CI	p-value
CDP	0.68	0.11-4.37	0.688
Treatment dose	0.98	0.93-1.04	0.577

CDP x treatment dose	1.01	0.91-1.12	0.829

Considering BPD as a covariate, there was again no difference between the model including BPD as a covariate and the basic model: $\chi^2(2) = 4.71$; $p = .095$ (Table 3).

Table 3 Regression model with BPD as covariate

Treatment response	OR	95% CI	p-value
CDP	0.65	0.20-2.07	0.466
BPD	3.15	0.34-29.53	0.315
CDP x BPD	1.43	0.09-23.95	0.804

In addition, we examined depression, assessed with the BDI-II (Table 4). Again, the regression model including depression as a covariate did not differ from the basic model: : $\chi^2(2) = 1.29$; $p = .524$.

Table 4 Regression model with depression (BDI-II) as covariate

Treatment response	OR	95% CI	p-value
CDP	0.21	0.01-5.65	0.353
BDI-II	0.99	0.92-1.07	0.805
CDP x BDI-II	1.05	0.95-1.15	0.370

<u>Control for confounding variables concerning NSSI frequency within the last month:</u>

In addition, we also examined the number of NSSI incidents within the last month at T0, T1 and T2 as a basic regression model. Examining treatment dose as a covariate, again likelihood-ratio tests showed that the model including treatment dose as a covariate did not differ from the basic model: $\chi^2(6) = 7.10$; $p = .312$ (Table 5).

Table 5 Regression model with treatment dose as covariate

Treatment response	IRR[a]	95% CI	p-value
T1	0.75	0.29-1.96	0.559
T2	0.29	0.10-0.80	0.018

CDP	1.05	0.30-3.72	0.937
T1xCDP	0.19	0.04-0.84	0.028
T2xCDP	0.18	0.04-0.82	0.027
Treatment dose	0.98	0.95-1.02	0.397
T1xTreatment dose	0.99	0.95-1.03	0.655
T2xTreatment dose	1.00	0.96-1.10	0.916
CDPxTreatment dose	1.03	0.96-1.10	0.466
T1xCDPxTreatment dose	1.04	0.95-1.13	0.394
T2xCDPxTreatment dose	1.06	0.98-1.15	0.164

a IRR=incidence rate ratio

When taking BPD into account, there was again no significant difference between the model including BPD as a covariate and the basic model:: $\chi^2(6) = 6.15$; $p = .407$ (Table 6).

Table 6 Regression model with BPD as covariate

Treatment response	IRR[a]	95% CI	p-value
T1	0.68	0.35-1.32	0.256
T2	0.30	0.15-0.61	0.001
CDP	1.21	0.49-3.00	0.685
T1xCDP	0.43	0.15-1.20	0.106
T2xCDP	0.34	0.11-1.01	0.052
BPD	1.08	0.30-3.84	0.911
T1xBPD	0.66	0.16-2.71	0.568
T2xBPD	0.90	0.21-3.88	0.892
CDPxBPD	1.93	0.37-10.05	0.434
T1xCDPxBPD	0.57	0.09-3.67	0.553
T2xCDPxBPD	1.54	0.22-10.57	0.662

In a further regression model, depression was examined as a covariate. Again, the model including depression as a covariate did not differ from the model without depression as a covariate: $\chi^2(18) = 24.43$; $p = .142$ (Table 7).

Table 7 Regression model with depression (BDI-II) as covariate

Treatment response	IRR	95% CI	p-value
T1	1.24	0.11-14.23	0.864
T2	2.21	0.23-21.13	0.492
CDP	3.49	0.19-64.73	0.402
T1xCDP	0.11	0.00-4.88	0.257
T2xCDP	0.06	0.00-2.43	0.138
BDI-II			
mild	5.90	0.14-255.64	0.356
moderate	7.10	0.63-79.91	0.112
acute	13.50	1.39-130.83	0.025
T1xBDI-II mild	3.16	0.07-149.41	0.558
T1xBDI-II moderate	0.89	0.06-13.16	0.930
T1xBDI-II acute	0.36	0.03-4.60	0.435
T2xBDI-II mild	2.02e-09	0-.	0.998
T2xBDI-II moderate	0.27	0.02-3.37	0.307
T2xBDI-II acute	0.09	0.01-0.98	0.048
CDPxBDI-II mild	0.09	0.00-21.47	0.385
CDPxBDI-II moderate	0.39	0.01-10.35	0.570
CDPxBDI-II acute	0.49	0.02-10.36	0.649
T1xCDPxBDI-II mild	4.84	0.01-2117.44	0.611
T1xCDPxBDI-II moderate	4.93	0.08-296.00	0.445
T1xCDPxBDI-II acute	2.49	0.05-120.34	0.645
T2xCDPxBDI-II mild	49.83	0-.	1.000
T2xCDPxBDI-II moderate	6.51	0.12-355.60	0.358
T2xCDPxBDI-II acute	7.70	0.18-336.85	0.290
